# A Multidimensional Tool Based on the eHealth Literacy Framework: Development and Initial Validity Testing of the eHealth Literacy Questionnaire (eHLQ)

**DOI:** 10.2196/jmir.8371

**Published:** 2018-02-12

**Authors:** Lars Kayser, Astrid Karnoe, Dorthe Furstrand, Roy Batterham, Karl Bang Christensen, Gerald Elsworth, Richard H Osborne

**Affiliations:** ^1^ Department of Public Health University of Copenhagen Copenhagen Denmark; ^2^ The Danish Multiple Sclerosis Society Valby Denmark; ^3^ Danish Cancer Research Center The Danish Cancer Society Copenhagen Denmark; ^4^ Health Systems Improvement Unit Centre for Population Health Research, Faculty of Health Deakin University Geelong Australia; ^5^ Faculty of Public Health Thammasat University Bangkok Thailand

**Keywords:** eHealth, health literacy, computer literacy, questionnaire design

## Abstract

**Background:**

For people to be able to access, understand, and benefit from the increasing digitalization of health services, it is critical that services are provided in a way that meets the user’s needs, resources, and competence.

**Objective:**

The objective of the study was to develop a questionnaire that captures the 7-dimensional eHealth Literacy Framework (eHLF).

**Methods:**

Draft items were created in parallel in English and Danish. The items were generated from 450 statements collected during the conceptual development of eHLF. In all, 57 items (7 to 9 items per scale) were generated and adjusted after cognitive testing. Items were tested in 475 people recruited from settings in which the scale was intended to be used (community and health care settings) and including people with a range of chronic conditions. Measurement properties were assessed using approaches from item response theory (IRT) and classical test theory (CTT) such as confirmatory factor analysis (CFA) and reliability using composite scale reliability (CSR); potential bias due to age and sex was evaluated using differential item functioning (DIF).

**Results:**

CFA confirmed the presence of the 7 a priori dimensions of eHLF. Following item analysis, a 35-item 7-scale questionnaire was constructed, covering (1) using technology to process health information (5 items, CSR=.84), (2) understanding of health concepts and language (5 items, CSR=.75), (3) ability to actively engage with digital services (5 items, CSR=.86), (4) feel safe and in control (5 items, CSR=.87), (5) motivated to engage with digital services (5 items, CSR=.84), (6) access to digital services that work (6 items, CSR=.77), and (7) digital services that suit individual needs (4 items, CSR=.85). A 7-factor CFA model, using small-variance priors for cross-loadings and residual correlations, had a satisfactory fit (posterior productive *P* value: .27, 95% CI for the difference between the observed and replicated chi-square values: −63.7 to 133.8). The CFA showed that all items loaded strongly on their respective factors. The IRT analysis showed that no items were found to have disordered thresholds. For most scales, discriminant validity was acceptable; however, 2 pairs of dimensions were highly correlated; dimensions 1 and 5 (*r*=.95), and dimensions 6 and 7 (*r*=.96). All dimensions were retained because of strong content differentiation and potential causal relationships between these dimensions. There is no evidence of DIF.

**Conclusions:**

The eHealth Literacy Questionnaire (eHLQ) is a multidimensional tool based on a well-defined a priori eHLF framework with robust properties. It has satisfactory evidence of construct validity and reliable measurement across a broad range of concepts (using both CTT and IRT traditions) in various groups. It is designed to be used to understand and evaluate people’s interaction with digital health services.

## Introduction

Modern health promotion and health care with increasing digitalization of information and services have become increasingly challenging for both community members and providers [1,2]. Community members can be delivered a panoply of messages from many media and may have access to information from a rapidly growing World Wide Web of information and service providers [3].

For people to be able to effectively and equitably access health services, it is critical that such services are provided in a way that generates appropriate actions and that the recipient benefits in the intended way. If people have a range of health literacy limitations, that is, limitations across “the cognitive and social skills which determine the motivation and ability of individuals to gain access to, understand and use information in ways which promote and maintain good health” [4], they are at risk of having reduced access to care, poor self-management skills, increased hospitalization, and decreased life span [5].

With the increasing digitalization of health care through electronic services, including health portals and health records, which are accessed by people from their homes, a new level of complexity has been added to the ways health care systems and the community have to interact.

The increased complexity demands a range of digital competencies among users, and this then calls for new ways to describe and evaluate users’ digital capabilities and experiences in this rapidly changing health context.

Consequently, the concept of eHealth literacy (or digital health literacy) has emerged. Norman and Skinner (2006) described it as “the ability to seek, find, understand, and appraise health information from electronic sources and apply the knowledge gained to addressing or solving a health problem” [6]. Norman and Skinner’s concept has since been updated by others to include elements related to users’ cognitive skills [7,8] and dimensions such as communication, cultural elements, and social elements [9]. Despite increasing interest in this eHealth literacy concept, limited evidence exists regarding whether addressing people’s eHealth literacy improves health outcomes [10]. This may be due to either a lack of appropriate instruments to measure eHealth literacy or, until now, low adoption of technology when providing health care [10,11].

In 2015, we identified a new concept for eHealth literacy: a model based on systematic and inductive methods that sought to identify the full range of elements relevant to individuals attempting to understand and use eHealth technologies and digital services [12].

**Figure 1:**
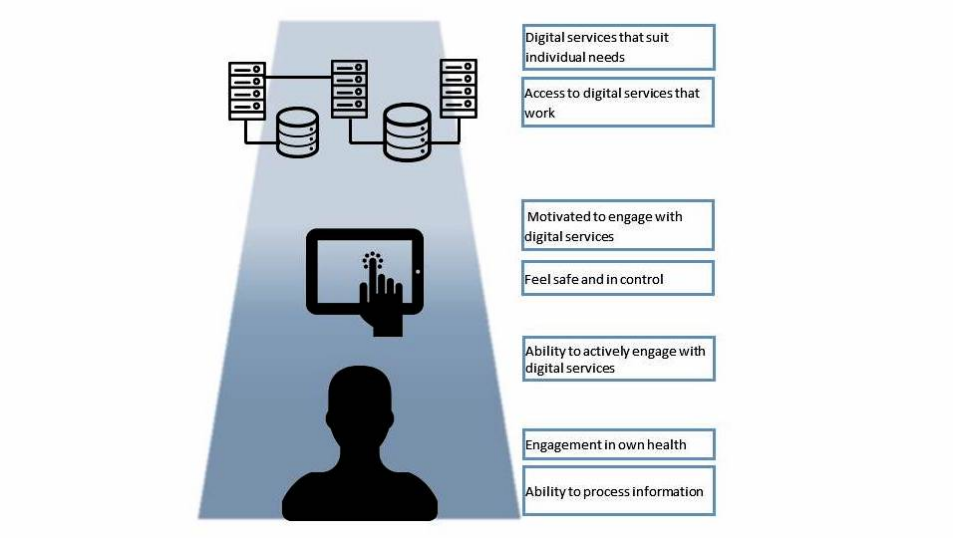
The eHealth literacy framework (eHLF).

This model, the eHealth Literacy Framework (eHLF), consists of 7 dimensions that describe the attributes of the users (information and knowledge about their health); the intersection between users and the technologies (their feeling of being safe and in control and their motivation); and users experience of systems (they work and are accessible, and suits users’ needs). The eHLF provides a comprehensive map of the individual technology user’s health literacy that covers his or her knowledge and skills, the system, and how the individual interacts with the system (see Figure 1).

The eHLF was specifically designed to inform the development of a conceptually and psychometrically sound questionnaire measure of eHealth literacy. The aim of this study was to create and test items and scales that capture the 7 eHLF dimensions using the validity-driven approach [13].

## Methods

### Initial Development

Development of the eHLQ followed the validity-driven approach [13] that has been used to develop several widely used and highly robust questionnaires [14-16]. The objective of the eHLQ development was to create an instrument that captures the 7 hypothesized dimensions of eHLF. Items were based on 450 statements obtained from Norgaard et al’s (2015) conceptual development of eHLF [12]. These statements had been collected during 8 concept mapping sessions, which included participation of patients (recruited from patient organizations and primary care clinics), computer scientists, academics, and health professionals [12]. Items for the questionnaire were simultaneously written in Danish and English as recommended by Eremenco (2005) [17] to avoid words or phrases that may be difficult to translate into other languages. To provide a rich range of candidate items for the construction of each scale, up to 9 items were drafted for each of the 7 eHLF dimensions. The statements were written to relate to a 4-point set of response options: strongly disagree, disagree, agree, and strongly agree, assigned a value of 1 to 4, respectively. Items were also written to represent a range of difficulty that were broadly guided by Bloom’s taxonomy (ie, related to knowledge and remembering, understanding, applying, analyzing, and evaluating) [18] so that the full range of the construct could be covered. The items were extensively discussed in Australian, Danish, and other contexts by the multidisciplinary team whose members have extensive experience in writing questionnaire items and applying them across cultures.

Danish draft items were tested in 7 cognitive interviews to check whether respondents understood the items as intended [19]. The cognitive testing involved initial administration of items using paper and pen format with careful observation of each respondent. The interviewer then reviewed items with the respondent and asked specific questions about items the respondent had hesitated on or appeared to have found difficult in answering. Respondents were asked “What were you thinking about when you were answering that question?” This process elicited the cognitive process behind the answers. A prompt was used if needed: “Why did you select that response option?” The items were adjusted based on the inputs with a particular focus on how the informants understood terms or concepts in relation to providers of health care and technologies.

### Recruitment of Participants

Respondents were recruited from a wide range of sociodemographic settings to broadly represent the targets for the application of the questionnaire in the future. Individuals were included if they were above age 18 years and able to read or understand Danish. Potential respondents were randomly approached by trained interviewers in a variety of locations in the broader community, such as in libraries, private sector workplaces, a hospital, nursing homes, health centers, and an outpatient clinic. To ensure inclusion of people who may have low literacy, potential respondents were given the option of completing the questionnaire themselves or to have it read aloud in an interview. If respondents did not have time to finish the questionnaire, they were encouraged to complete it at home and were provided with a reply-paid envelope. They also had the option of completing a Web-based questionnaire.

Demographic data including age, sex, educational background, self-reported health condition, and presence of chronic conditions were also collected to evaluate whether the resulting scales were invariant to these exogenous factors and thus provided unbiased estimates of mean differences across these groups.

The administration of the questionnaires also included the administration of a validation version of the eHealth literacy assessment toolkit, which is reported elsewhere (personal communication, Karnoe 2017). Respondents did not receive any payment for filling out the questionnaire.

### Item Analysis and Selection

The first step in the analysis for the items was to examine item characteristics. It was intended that each scale would have the smallest number of items necessary to capture the meaning of the construct in the most efficient manner while ensuring adequate coverage of the construct. Each item and each set of items forming a scale, as well as associations between and across items and scales, were tested using psychometric procedures afforded by the conventions of both classical test theory [20] and item response theory (IRT) [21].

### Statistical Analysis

Descriptive statistics were generated for each item to determine missing values, floor and ceiling effects, interitem correlations, correlation with scale score, scale reliability (composite reliability, Cronbach alpha, person separation index), estimated item location, and *P* value for item fit tests. Given that hypothesized constructs were specified a priori within the eHLF [12], confirmatory factor analysis (CFA) was used.

Results from each of these analyses were used to assist with decisions about optimizing the number of items in each scale. Of central importance to the item deletion retention strategy was ensuring that the retained items properly represented the intended a priori construct from the eHLF. Items that performed poorly on the above criteria were earmarked for deletion. We also sought to generate scales that had a minimum reliability of .8 but where items had no excessively high interscale correlations, violations of local independence, or high correlated residuals. Where the content of items had substantial overlap, and tended to perform well on a range of indicators, the item selection strategy then included criteria to improve the diversity of item locations, that is, selection of a range of items within a scale that range across all difficulty levels of the construct that the scale measures.

CFA was conducted with Mplus versions 7.4 and 8 (Muthén & Muthén, Los Angeles, CA, USA) using Bayesian Structural Equation Modeling (BSEM) [22,23]. BSEM is a specific application of Bayesian statistical analysis to factor analysis and structural equation modeling. In a conventional CFA usage, for example, maximum likelihood analysis, model parameters that represent possible cross-loadings and correlations between item residuals are typically set to be exactly zero. When these restrictions are placed on CFA models used to confirm, a multifactor structure poor model fit often results, and a large number of stepwise modifications (estimation of parameters originally constrained to zero) can be required to achieve a reasonably fitting result. This approach may not, however, necessarily lead to a single solution depending on the sequence of choices of modifications made along the way, and there is frequently a strong upward bias in the estimation of interfactor correlations, leading to erroneous conclusions about scale discriminant validity. By using small-variance priors, BSEM allows models to be fitted that have the flexibility to estimate small variations from these strictly zero constraints. For the final BSEM analysis, after a little systematic experimentation (Multimedia Appendix 1), the variance of the Bayesian priors for the cross-loadings was set at 0.01 such that there was a 95% probability that the cross-loadings would be within the range ±.20. Similarly, the variance for the residual correlations was set to give a 95% probability that the correlations were within the range of ±.2 [22].

The approach to model fit in BSEM differs from that in conventional CFA. The conventional CFA fit indices (eg, chi-square, comparative fit index, and root mean square error of approximation) are not used. Rather, fit in BSEM is assessed using a procedure called “posterior predictive checking” that results in a “posterior predictive *P* value” (PPP value). In a very well-fitting model, the PPP value is expected to be close to .5, whereas a value of <.05 (or <.10 or <.01 if less or greater stringency is applied) is typically regarded as indicating unsatisfactory fit. A PPP value of >.05 was chosen as the threshold for satisfactory fit in this study. Additionally, a fit statistic derived from the Bayesian model-fitting process is calculated with 95% CIs. A pattern of symmetrical upper and lower CIs centered around zero indicates excellent fit, whereas a lower 95% CI that is positive suggests that the fit is not satisfactory [22-24].

The IRT model-based evaluation of item fit used a unidimensional IRT model—the generalized partial credit model (GPCM) [25]. For each scale, we compared the observed item scores with the scores expected under the model using an implementation in SAS statistical software version 9.4 (SAS Institute, Cary, NC, USA) [26]. This provides a graphical test of item fit where the item’s mean score is plotted against the group’s scale mean and evaluated against values.

For each item, the estimated item discrimination parameter (the IRT-equivalent of a factor loading) and the estimated item location (computed as the average of the item thresholds) were reported.

Differential item functioning (DIF) is a statistical characteristic of an item indicating the extent to which the item can be said to measure the same construct across subpopulations [27]. We tested DIF using logistic regression techniques [28-30] addressing the elevated risk of type I error due to the larger number of tests performed and by the Benjamini and Hochberg procedure [31] controlling the false discovery rate results.

### Ethical Considerations

According to Danish law, when survey-based studies are undertaken in accordance with the Helsinki Declaration, specific approval by an ethics committee and written informed consent are not required. Potential respondents were provided with information about the survey and its purpose, including that participation was voluntary. The completion of the survey by participants was then considered to be implied consent.

## Results

### Item Construction and Refinement

Between 7 and 9 draft items per scale were generated (58 items in total). Cognitive testing was undertaken with 7 individuals (4 women), aged between 16 and 74 years from different cultural backgrounds and with varying educational levels. The interviews found that almost all items were understood as intended; 1 item was removed due to unclear text, whereas only some small refinements were made to other items to improve clarity and simplicity. The refinements mainly related to getting the clearest possible Danish words related to the core concept of technology, digital tools, or electronic tools. The term “digital” was preferred across demographic groups. Moreover, the best Danish term to express “health” and “health conditions” was tested, and the term “health” was found to work the best. The final number of items for going to the field was 57.

### Item Number Reduction and Scale Construction

The refined items were randomized and administered to 475 individuals—100 outpatients who all filled in a paper version. Out of the 375 people in the community a total of 328 filled in the paper version and 47 filled in the digital version.

The hypothesized 7-factor BSEM model for the initial 57 items showed a satisfactory fit to the data (PPP value .79, 95% CI for the difference between the observed and replicated chi-square values −239.75 to 99.85). A total of 10 items had low (<.5) standardized factor loadings; however, 3 items showed evidence of factorial complexity having cross-loadings >.25. There were also 15 pairs of items with residual correlations >.30. Of these item pairs, 12 correlated residuals were between items that were associated with different target factors, suggesting that, from an IRT perspective, the assumption of local independence of the hypothesized scales was largely satisfied.

After inspection of the results of the initial BSEM analysis and the parallel IRT analyses, items that performed poorly or were clearly redundant to others (ie, had highly similar content and measurement properties) across analyses were iteratively removed, resulting in 35 items in 7 scales comprising 4, 5, or 6 items in each (see Multimedia Appendix 2).

A final BSEM model (Multimedia Appendix 1) was then fitted to the data for these items. Model fit was satisfactory (PPP value .27, 95% CI for the difference between the observed and replicated chi-square values −63.7 to 133.8). With 4 exceptions, all factor loadings were >.50 (see Multimedia Appendix 3). All loadings <.5 were >.4.

There were no statistically significant cross-loadings, and there were 8 residual correlations ≥.3. All but one of these larger residual correlations was negative with 4 associated with scale (1) using technology to process health information. The only positive residual correlation ≥.3 was between 2 contiguous items, former 44 and 45. They were, therefore, separated and ended up in the final version as item numbers 26 and 35. There were no within-scale positive residual correlations ≥.3.

The fit of the GPCM to the data was excellent in all of the dimensions (Multimedia Appendix 4).

Estimates of composite scale reliability are also shown in Multimedia Appendix 3 (95% CIs in parentheses, and, for comparison, Cronbach alpha) for the final 7 scales. All 7 scales had acceptable composite reliability (>.7), and 5 scales have a reliability above .8; however, scales 2 and 6 had somewhat lower reliability than intended (.75 and .77, respectively).

Interfactor correlations ranged from .31 (factors 3 and 4) to .97 (factors 6 and 7) with the next highest being .96 (factors 1 and 5), suggesting satisfactory discrimination between the majority of the scales with the exception of the following: (6) access to digital services that work; (7) digital services that suit individual needs; (1) using technology to process health information; and (5) motivated to engage with digital services (Table 1).

The DIF analysis showed no evidence of influence of age or sex on the item scores. The item locations, item discriminations, and factor loadings are shown in Multimedia Appendix 3. As expected, the order of factor loadings and the order of the item discriminations are very similar. The spread in the item locations indicate that, for each scale, the items represent a range of difficulty, indicating that the full range of the construct is covered. The analyses generated scales that were well targeted to the respondent sample (Table 1) and showed good distributional properties (Table 2).

An analysis of the construct representation, that is, the completeness of the match between the intended construct (first column in Multimedia Appendix 2) and the content of the resulting scales (second column, Multimedia Appendix 3) indicated strong concordance between the intended and generated constructs for 5 of the 7 constructs. For construct 1, ability to process information, the specific elements of fundamental reading, writing, and cognitive ability were missing; however, they were represented through higher-order functions such as application and use of such skills. This scale was renamed as (1) using technology to process health information. For construct 2, engagement in own health, the surviving items focused more on whether the respondents perceived that they had adequate knowledge and understanding of their health in general terms. The intended construct of eHLF had a broader focus on awareness and engagement in and utilization of information and knowledge about health.

**Table 1:** Interfactor correlation between the eHealth Literacy Questionnaire scales.

Factor name	Factor 1	Factor 2	Factor 3	Factor 4	Factor 5	Factor 6
Factor 2. Understanding of health concepts and language	0.69					
Factor 3. Ability to actively engage with digital services	0.90	0.61				
Factor 4. Feel safe and in control	0.36	0.49	0.31			
Factor 5. Motivated to engage with digital services	0.95	0.61	0.83	0.47		
Factor 6. Access to digital services that work	0.77	0.57	0.73	0.69	0.83	
Factor 7. Digital services that suit individual needs	0.78	0.45	0.74	0.58	0.84	0.96

**Table 2:** eHealth Literacy Questionnaire scales and descriptive statistics.

No	Scale	n (%) (N=475)	Mean (SD)	Median (IQR^a^)
1	Using technology to process health information	462 (97.3)	2.55 (0.66)	2.60 (2.20-3.00)
2	Understanding of health concepts and language	466 (98.1)	2.97 (0.55)	3.00 (2.60-3.40)
3	Ability to actively engage with digital services	465 (97.9)	2.81 (0.69)	2.80 (2.40-3.20)
4	Feel safe and in control	466 (98.1)	2.61 (0.66)	2.60 (2.20-3.00)
5	Motivated to engage with digital services	466 (98.1)	2.55 (0.65)	2.60 (2.00-3.00)
6	Access to digital services that work	466 (98.1)	2.52 (0.55)	2.50 (2.17-2.83)
7	Digital services that suit individual needs	457 (96.2)	2.42 (0.62)	2.33 (2.00-3.00)

^a^IQR: interquartile range.

The construct included, potentially, a very wide range of elements around physiological functions, risk factors, and elements of the health care system. This disparate range of elements would require an inventory to capture in complete breadth of the construct; however, the eHLQ items generated and surviving the item reduction phase, captured a somewhat higher order assessment of the respondent’s understanding and engagement in health information, which is more suitable for a psychometric scale, rather than an inventory. The scale was renamed as (2) understanding of health concepts and language.

## Discussion

### Principal Findings

We sought to develop and test a new measure of eHealth literacy using both classical and modern psychometric approaches to questionnaire development. Using a robust conceptual model, developed through extensive local and international community consultation [12] and through a validity-driven approach [12,13], 7 new psychometrically robust scales to comprehensively measure the broad concept of eHealth literacy were developed. Construction and validity testing in a broad range of target groups generated clear evidence of construct validity, discriminant validity, and scale reliability. This initial validity testing indicates that the eHLQ is likely to be valuable for the characterization and understanding of digital health literacy.

This research introduced some highly rigorous and innovative elements to questionnaire development and validation. First, the data to generate the eHLF model were obtained using concept mapping in 2 cultures (Danish and English) and through an international e-consultation. Concept mapping has been found to be a highly robust technique for developing conceptual models and for questionnaire development [32]. Additionally, when the items were written, they were simultaneously constructed in Danish and English. With this step, we sought to uncover and remove idiomatic expressions in either language so that when it is translated to further languages, only concrete concepts are presented for translation with fidelity and to support international equivalence of the constructs. We also employed extensive and rigorous application of the two, often opposing, traditions of psychometric analysis: CTT and IRT. The items surviving the scrutiny of these procedures, as well as from rigorous qualitative methods, have satisfactory psychometric properties in all the scales.

The eHLQ is now ready for application, and for further testing, in a wide range of settings and purposes; these include the following:

- Evaluation of interventions. The eHLQ will provide insight into users’ perceptions and experiences when using digital health solutions. As with previous tools developed using the same methods as the eHLQ [14,15], we expect that, with further testing, it will be a robust patient-reported outcome measure and sensitive to change.
- Implementation and adoption of digital health services. We also expect the scales to provide insights into why digital health services implementations work or fail (ie, understanding intermediate or process outcomes). Given that the scales cover individual user attributes, attributes of the system, and the interaction between the two, the eHLQ is expected to uncover mechanisms that determine adoption outcomes.
- Community and population surveys. The eHLQ is expected to be useful for local, regional, and populations surveys. This information will inform policy makers, program managers, and service providers about the profile of needs and strengths of individuals across the population and demographic subgroups.

The eHLQ also offers an opportunity to stratify users for inclusion in design processes [33] to explore the reactions of these groups to new digital solutions or, more generally, to document the strengths and weaknesses among user subgroups. Information about subgroups with respect to their eHLQ profile (of strengths and weaknesses) and other characteristics may be used to design interventions or inform designers through creation of archetypes or personas as described the in Ophelia (OPtimising HEalth LIteracy and Access) process developed for service redesign and development of person-centered services [34,35].

A further important element of scale construction was the consideration of within-construct concept representation and item difficulty. The items we drafted sought to cover the full range of issues within a construct and to achieve a spread of item difficulties from items being easy to answer to items being hard to score highly. The statistical analyses demonstrate that these demanding targets were broadly achieved. Some subconcepts identified in the concept mapping, and detailed in the eHLF, did not survive the item development and testing process, and therefore, some scales are not as broad as initially intended. The requirement for being faithful to the codesign outcomes (the eHLF), broad item difficulties, and meeting the requirements of the 2 psychometric traditions has generated a tool that robustly captures the concept of eHealth literacy, but with some minor subcomponents underrepresented. If researchers and developers wish to capture the omitted subelements (basic functional health literacy or broader issues around knowledge and engagement), other tools should be used to supplement the eHLQ, such as the eHealth literacy assessment toolkit (personal communication, Karnoe 2017), or the digital health literacy instrument (DHLI) by van der Vaart [36].

We found that there are 2 particularly high interfactor correlations between factors 1 and 5 (*r*=.95) and factors 6 and 7 (*r*=.96). The eHLF contains 7 dimensions, all of which we sought to include in the final eHLQ model. Content analysis, the careful deliberations while developing items, and the original views of patient and professional groups indicated that each of the original constructs were different. The high correlations may be a result of them being located along the same causal path. When considering the content of these sales, we expect that different interventions will result in different patterns of change in the pairs of related scales. This should be carefully monitored over time and across different settings.

The psychometric and construct representation demands we placed on the eHLQ construction process were further compounded by the need for the eHLQ to be a relatively short questionnaire. Importantly, all of the scales have acceptable reliability, despite having only 4 to 6 items. Given that the eHLQ is intended for application among people with low literacy and they may be ill, it is critical that the smallest possible number of items be included. Every scale had satisfactory loadings on its intended items, with negligible cross-loadings on other items. Although we had hoped all scales would have a reliability ˃.8, this was not quite achieved for 2 scales (≥.75 for both). Importantly, this level of reliability is acceptable for research and evaluation purposes.

Future concurrent validity and other validity tests, including predictive validity tests, will be valuable. For the most part, we have found that the concept mapping, and subsequent qualitative studies, ensures that the validity of the data the questionnaire generates is robust. The eHEALS [37] appears to be the best current measure of some elements of eHealth literacy, and it will be valuable for future researchers to compare the tools.

Moreover, future research should include further quantitative and qualitative work to develop interpretation and use arguments [38] to generate comprehensive validity testing of the eHLQ across a wide range of contexts to support current and future users understand the data generated by the eHLQ.

The eHLQ provides a wider range of dimensions of eHealth literacy than previous tools. It covers not only an individual’s competences, as in the Lily model [6] and the van der Vaart DHLI model [36], but also an individual’s experiences and interactions with technologies and services. It provides a much richer understanding of the whole of the system through providing perspectives of individuals, the system, and the interaction between the two.

Recent study by van der Vaart [36] specifically expanded the digital health field by creating 7 areas that mainly focus on individual competencies. These are operational skills, navigation skills, information searching, evaluating reliability, determining relevance, adding self-generated content, and protecting privacy.

The introduction of the eHLQ’s scales covering user interaction and the user’s experience of engaging with the system is an important innovation for the rapidly growing digital health field. The eHLQ has the potential to provide insight into the maturity of a country’s digital services. With mature digital health services, we expect that individuals find the system more responsive to their needs, and thus can engage more fully in support of achieving health and equity. This is akin to the new health concept of health literacy responsiveness [39]. We expect that in a digitally mature society, the scales covering the system and the interaction will reflect stronger: (6) access to digital services that work and (7) digital services that suit individual needs. In settings with developing systems, where there are few digital services or limited or piecemeal coverage and access, the scores on these scales are expected to reflect major challenges for individuals.

It is important to note that eHealth literacy, like the concept of health literacy, is both a reflection of the individual’s knowledge and the skills he or she may employ in the cultural, social, and institutional context in which they are engaging in; therefore, it is critical to assess these domains simultaneously [9,12,40]. As we see wide-scale improvements in digital health services, we would expect to see the user interaction scales improve—that is, (4) feel safe and in control and (5) motivated to engage with digital services.

The eHLQ has already generated substantial interest in the field. It is currently being translated into Chinese, Norwegian, and Czech, and a German-speaking country initiative is under way. The English version is also undergoing validity testing. The conceptual model and the range of intended applications fit well with a wide range of current policy initiatives. These include the World Health Organisation (WHO) People Centred Health Services Framework [1] and the WHO Shanghai Declaration, which places health literacy as one of the three key elements to support the achievement of the sustainable development goals by 2030 [41]. To reach these ambitious goals, eHealth literacy responsive systems need to be implemented on a wide scale to leave no one behind. The eHLQ is currently in the field and is available with the authors.

### Conclusions

The eHLQ is a psychometrically robust multidimensional instrument with 7 scales that comprehensively cover all 7 dimensions of the eHLF. The eHLQ and the eHLF’s conceptual underpinnings are likely to be a useful set of tools to support researchers, developers, designers, and governments to develop, implement, and evaluate effective digital health interventions.

## Appendix

Multimedia Appendix 1 Fit statistics and summary model parameters for six Bayesian structural equation models with varying prior variances for cross-loadings and correlated residuals–model 3 chosen as a satisfactory solution.

Multimedia Appendix 2 The 7 constructs of the eHealth Literacy Framework and derived scales of the eHealth Literacy Questionnaire.

Multimedia Appendix 3 The estimated item locations, item discriminations, and factor loadings.

Multimedia Appendix 4 Illustration of the fit of items applying item response theory and the generalized partial credit model. The observed items scores are compared with the scores expected under the model.

Multimedia Appendix 5 eHLQ Licence Agreement.

## Abbreviations

BSEM: Bayesian Structural Equation Modeling
CFA: confirmatory factor analysis
CSR: composite scale reliability
CTT: classical test theory
DHLI: digital health literacy instrument
DIF: differential item functioning
eHLF: eHealth Literacy Framework
eHLQ: eHealth Literacy Questionnaire
GPCM: generalized partial credit model
IRT: item response theory
Ophelia: OPtimising HEalth LIteracy and Access
PPP: posterior predictive *P* value
