# Supplementary material for: A Multidimensional Tool Based on the eHealth Literacy Framework: Development and Initial Validity Testing of the eHealth Literacy Questionnaire (eHLQ)
Source: J Med Internet Res. 2018 Feb 12;20(2):e36. doi: 10.2196/jmir.8371 (PMC5826975; doi:10.2196/jmir.8371)
Supplement: Multimedia Appendix 1 [file jmir_v20i2e36_app1.pdf]

Multi-media Appendix 1

Fit statistics and summary model parameters for six Bayesian Structural Equation models with varying prior variances for cross-loadings and correlated residuals – Model 3 chosen as a satisfactory solution

Model parameter	Model 1 0.01/300*	Model 2 0.02/300	Model 3 0.01/200	Model 4 0.02/200	Model 5 0.01/100	Model 6 0.02/100
Posterior Predictive P Value (PPP)	0.058	0.084	0.265	0.304	0.615	0.634
95% CIs for X^2 Difference	-20.39 – 190.22	-30.22 – 180.61	-68.68 – 133.82	-75.02 – 127.32	-117.22 – 85.98	-121.81 – 81.58
Prior-posterior P-value (PPPP)			0.782	0.861		
DIC	32,782.87	32,761.43	32,765.87	32,738.70	32,773.99	32,750.71
N iterations to PSR consistently <1.05	9,900	10,100	4,900	5,000	3,800	10,300
N iterations to PSR consistently <1.01	14,000	14,900	18,500	20,000+	20,000+	20,000+
N (%) of sig. target loadings	35 (100%)	35 (100%)	35 (100%)	35 (100%)	35 (100%)	35 (100%)
N of sig. cross-loadings	0	0	0	0	0	0
N of sig. correlated residuals	30	25	37	31	51	
Range of inter-factor correlations	0.30 - 0.96	0.28 – 0.96	0.31 - 0.95	0.29 - 0.95	0.34– 0.95	

PPP = Posterior Predictive P Value; PPPP = Prior-posterior Predictive P-value; PSR = Potential Scale Reduction

- = variance of prior for cross-loadings / Inverse Wishart degrees of freedom for residual correlations
