# Supplementary material for: A Multidimensional Tool Based on the eHealth Literacy Framework: Development and Initial Validity Testing of the eHealth Literacy Questionnaire (eHLQ)
Source: J Med Internet Res. 2018 Feb 12;20(2):e36. doi: 10.2196/jmir.8371 (PMC5826975; doi:10.2196/jmir.8371)
Supplement: Multimedia Appendix 2 [file jmir_v20i2e36_app2.pdf]

Multimedia Appendix 2: The 7 constructs of the eHealth Literacy Framework and derived scales of the eHealth Literacy Questionnaire.

eHealth Literacy Framework constructs		eHealth Literacy Questionnaire scale names and items[a]	
1. Ability to process information		1. Using technology to process health information	
	Able to read, write and remember, apply basic numerical concepts, and understand context-specific language (eg, health, IT or English) as well as critically appraise information. Know when, how and what information to use		*I use technology to find...*
			I often use technology to understand...
			Technology helps me decide...
			I use technology to share...
			I use technology to organize...
2. Engagement in own health		2. Understanding of health concepts and language	
	Know about basic physiological functions and own current health status. Aware of risk factors and how to avoid them or reduce their influence on own health as well as navigating the health care system		*The information I have helps me...*
			I have enough information to take part in...
			I understand medical results...
			Overall, I understand how my body...
			I use measurements about my body to...
3. Ability to actively engage with digital services		3. Ability to actively engage with digital services	
	Being comfortable using digital services for handling information		*I know how to get...*
			I know how to make health technology...
			I can enter data...
			I quickly learn how to...
			I easily learn to use...
4. Feel safe and in control		4. Feel safe and in control	
	Feel that you have the ownership of personal data stored in the systems and that the data are safe and can be accessed only		*I am sure that my health data...*
			My electronic health care data are being stored...

	by people to whom they are relevant (own doctor, own nurse, etc)		*I have a clear understanding of...*
			I am sure that only authorized people...
			I am confident that health care providers...
5. Motivated to engage with digital services		5. Motivated to engage with digital services	
	Feel that engaging in the use of digital services will be useful for them in managing their health		*Technology makes me feel actively...*
			I find technology helps me...
			I find I get better services...
			Technology improves my communication...
			I find technology useful...
6. Access to digital services that work		6. Access to digital services that work	
	Have access to digital services that the users trust to be working when they need it and as they expect it to work		*Information about my health is always available...*
			My health care providers deliver services that I can access...
			My health data are available...
			All the health technology I use works...
			Most of my health care providers can be accessed...
			I have access to health technology that...
7. Digital services that suit individual needs		7. Digital services that suit individual needs	
	Have access to digital services that suit the specific needs and preferences of the users. This includes responsive features of both IT and the health care system (including careers) as well as adaptation of devices and interfaces to be used by people with physical and mental disabilities		*I find that digital services can adapt...*
			I find that digital health services seem to...
			I find digital health services are provided to me in a way...
			Digital health services provide me with easy ways...

[a]Items are truncated. The full list of items is available with the authors.
