# Supplementary material for: A Multidimensional Tool Based on the eHealth Literacy Framework: Development and Initial Validity Testing of the eHealth Literacy Questionnaire (eHLQ)
Source: J Med Internet Res. 2018 Feb 12;20(2):e36. doi: 10.2196/jmir.8371 (PMC5826975; doi:10.2196/jmir.8371)
Supplement: Multimedia Appendix 3 [file jmir_v20i2e36_app3.pdf]

Multimedia Appendix 3:

The estimated item locations, item discriminations, and factor loadings.

Item number		Item location (95% CI)	Factor loading	Item discrimination (95% CI)
1. *Using technology to process health information*				
7		2.04 (1.61-2.46)	*0.77*	2.12 (1.61-2.64)
11		0.69 (0.32-1.05)	*0.80*	2.33 (1.73-2.93)
13		0.25 (−0.14 to 0.64)	*0.64*	2.07 (1.59-2.56)
20		−2.16 (−2.79 to−1.53)	*0.52*	1.07 (0.81-1.32)
25		−0.97 (−1.43 to −0.51)	*0.57*	1.53 (1.17-1.88)
	Composite scale reliability (95% CIs) 0.84 (0.81-0.87)			
	Cronbach alpha .84			
2. *Understanding of health concepts and language*				
5		2.35 (1.73-2.98)	*0.60*	1.36 (1.01-1.71)
12		2.37 (1.89-2.84)	*0.80*	2.68 (1.86-3.50)
15		1.53 (1.05-2.01)	*0.62*	1.79 (1.32-2.26)
21		3.27 (2.54-4.01)	*0.60*	1.45 (1.08-1.83)
26		2.31 (1.61-3.01)	*0.43*	1.03 (0.75-1.31)
	Composite scale reliability (95% CIs) 0.75 (0.75-0.82)			
	Cronbach alpha .77			
3. *Ability to actively engage with digital services*				
4		1.88 (1.43- 2.33)	*0.76*	2.07 (1.59-2.55)
6		1.57 (1.18-1.96)	*0.84*	3.07 (2.29-3.86)
8		0.51 (-0.02-1.03)	*0.51*	0.93 (0.70-1.15)
17		1.34 (0.94-1.74)	*0.78*	2.61 (1.97-3.24)
32		0.54 (0.12-0.97)	*0.60*	1.97 (1.51-2.43)
	Composite scale reliability (95% CIs) 0.86 (0.83-0.88)			
	Cronbach alpha .86			
4. *Feel safe and in control*				
1		0.90 (0.51-1.29)	*0.81*	2.49 (1.92-3.06)
10		0.40 (0.04-0.76)	*0.83*	3.47 (2.58-4.35)
14		−0.78 (−1.34 to	*0.43*	1.05 (0.80-1.29)

		−0.23)		
22		0.12 (−0.25 to 0.49)	*0.82*	2.56 (1.96-3.15)
30		0.97 (0.55-1.39)	*0.71*	2.10 (1.63-2.58)
	Composite scale reliability (95% CIs) 0.87 (0.84-0.89)			
	Cronbach alpha .86			
5. *Motivated to engage with digital services*				
2		0.13 (−0.37 to 0.62)	0.68	1.13 (0.86-1.40)
19		−0.16 (−0.56 to 0.29)	0.79	2.19 (1.64-2.75)
24		−0.50 (−0.92 to −0.75)	0.67	2.17 (1.65-2.69)
27		0.20 (−0.20 to 0.59)	0.63	2.38 (1.76-2.99)
35		1.05 (0.61-1.49)	0.71	1.86 (1.41-2.30)
	Composite scale reliability (95% CIs) 0.84 (0.81-0.87)			
	Cronbach alpha .85			
6. *Access to digital services that work*				
3		2.16 (1.14-3.17)	*0.59*	0.55 (0.37-0.73)
9		0.21 (−0.30 to 0.72)	*0.44*	1.20 (0.89-1.50)
16		0.23 (−0.35 to 0.81)	*0.43*	0.86 (0.63-1.09)
23		−3.43 (−4.53 to −2.32)	*0.54*	0.79 (0.56-1.02)
29		−0.47 (−0.92 to −0.01)	*0.65*	1.91 (1.43-2.39)
34		0.16 (−0.21 to 0.53)	*0.75*	3.22 (2.15-4.29)
	Composite scale reliability (95% CIs) 0.77 (0.73-0.81)			
	Cronbach alpha .77			
7. *Digital services that suit individual needs*				
18		−1.00 (−1.46 to −0.55)	*0.75*	2.01 (1.54-2.49)
28		−1.10 (−1.53 to −0.67)	*0.79*	3.14 (2.32-3.96)
31		−0.15 (−0.57 to 0.27)	*0.72*	2.64 (2.00-3.29)
33		0.17 (−0.23 to 0.56)	*0.75*	2.45 (1.82-3.07)
	Composite scale reliability (95% CIs) 0.85 (0.82-0.88)			
	Cronbach alpha .85			
