# Supplementary material for: A Multidimensional Tool Based on the eHealth Literacy Framework: Development and Initial Validity Testing of the eHealth Literacy Questionnaire (eHLQ)
Source: J Med Internet Res. 2018 Feb 12;20(2):e36. doi: 10.2196/jmir.8371 (PMC5826975; doi:10.2196/jmir.8371)

1. Using technology process health information

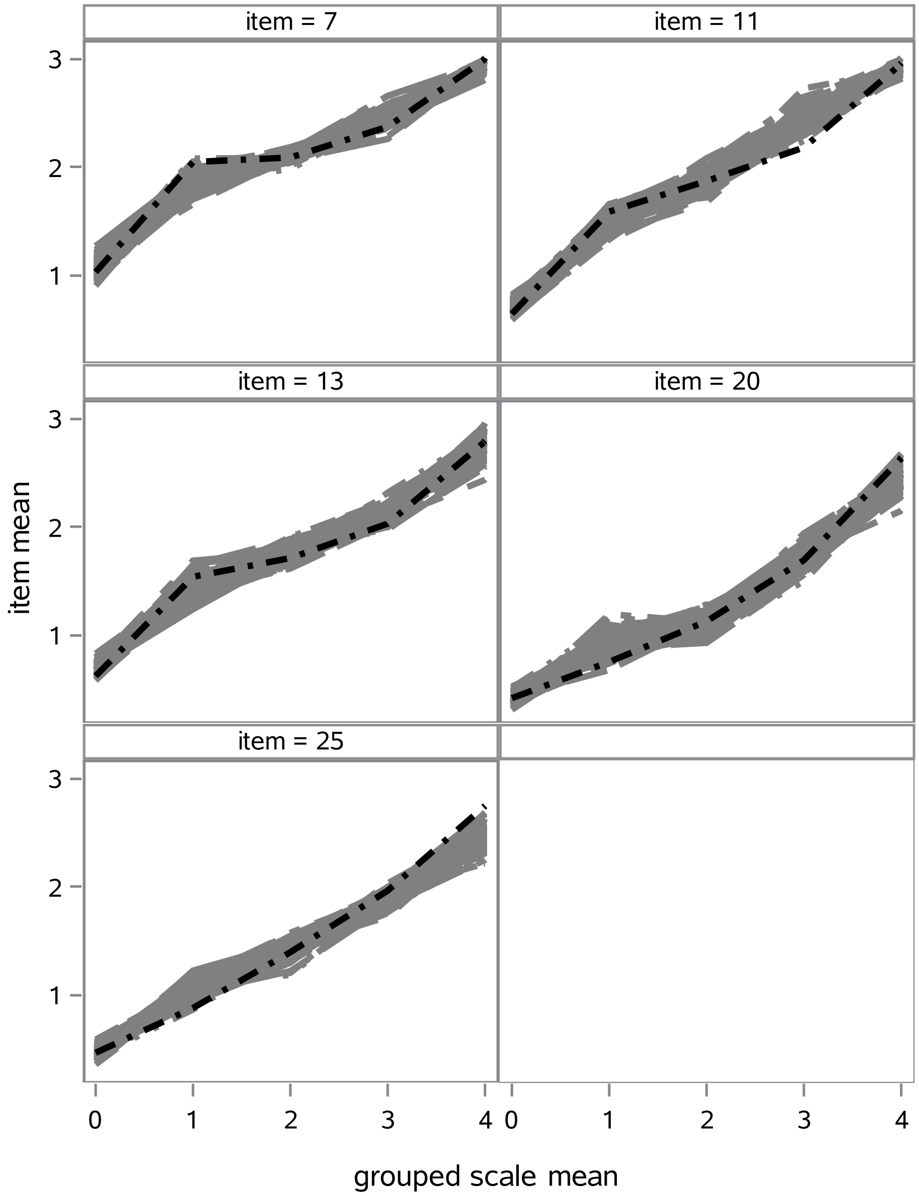

2. Understanding of health concepts and language

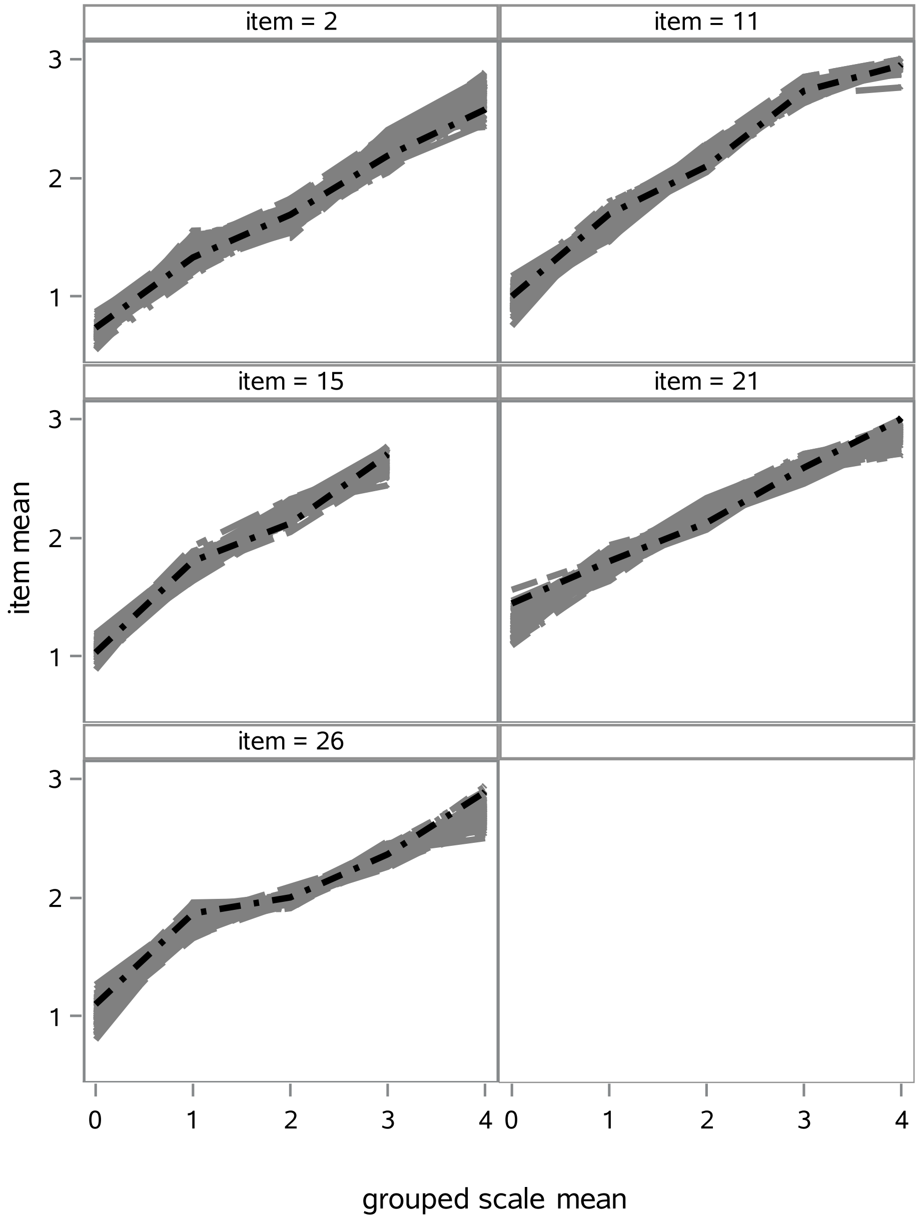

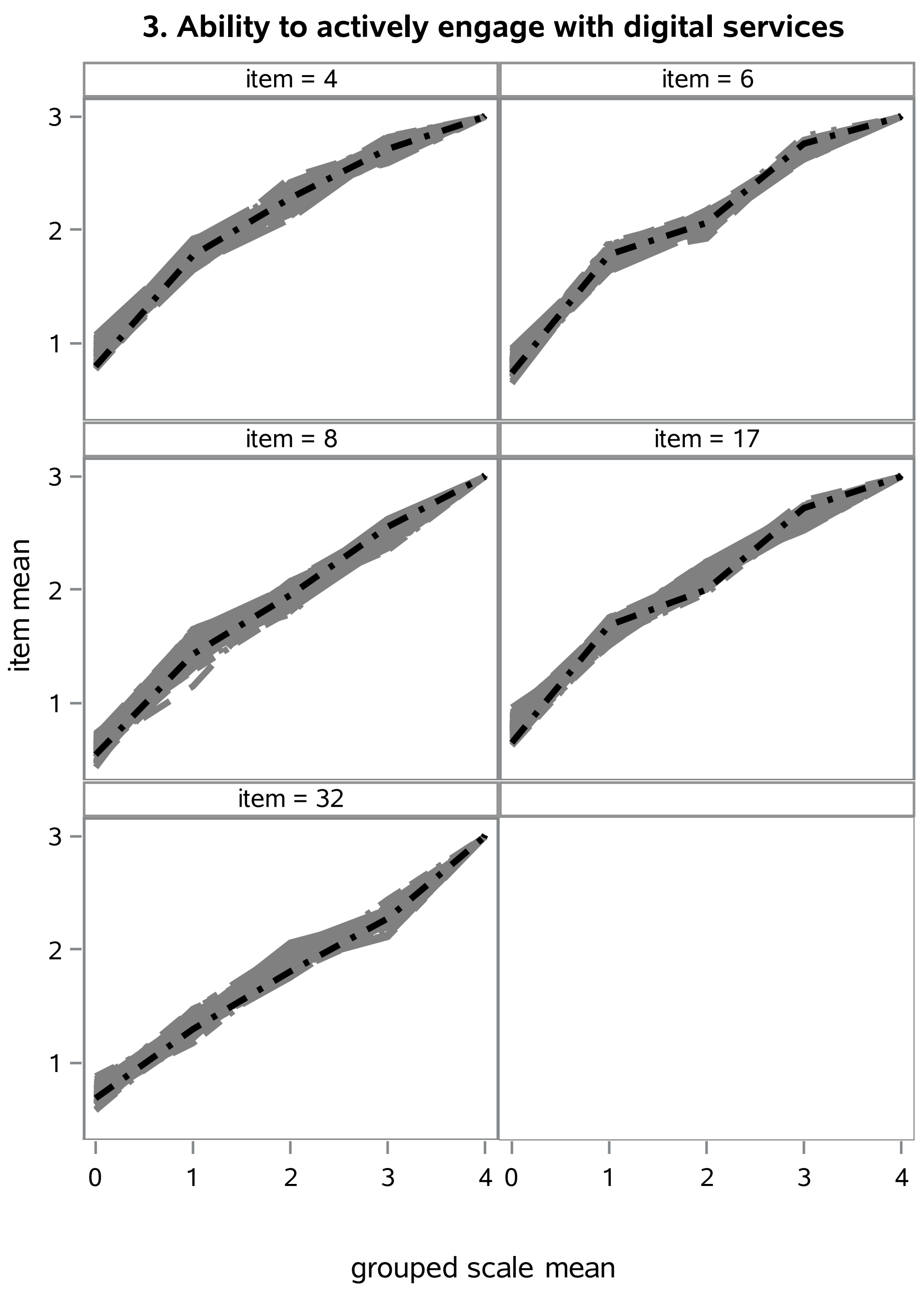

3. Ability to actively engage with digital services
item = 4
item = 6
item = 8
item = 17
item = 32
item mean
grouped scale mean
0
1
2
3
4

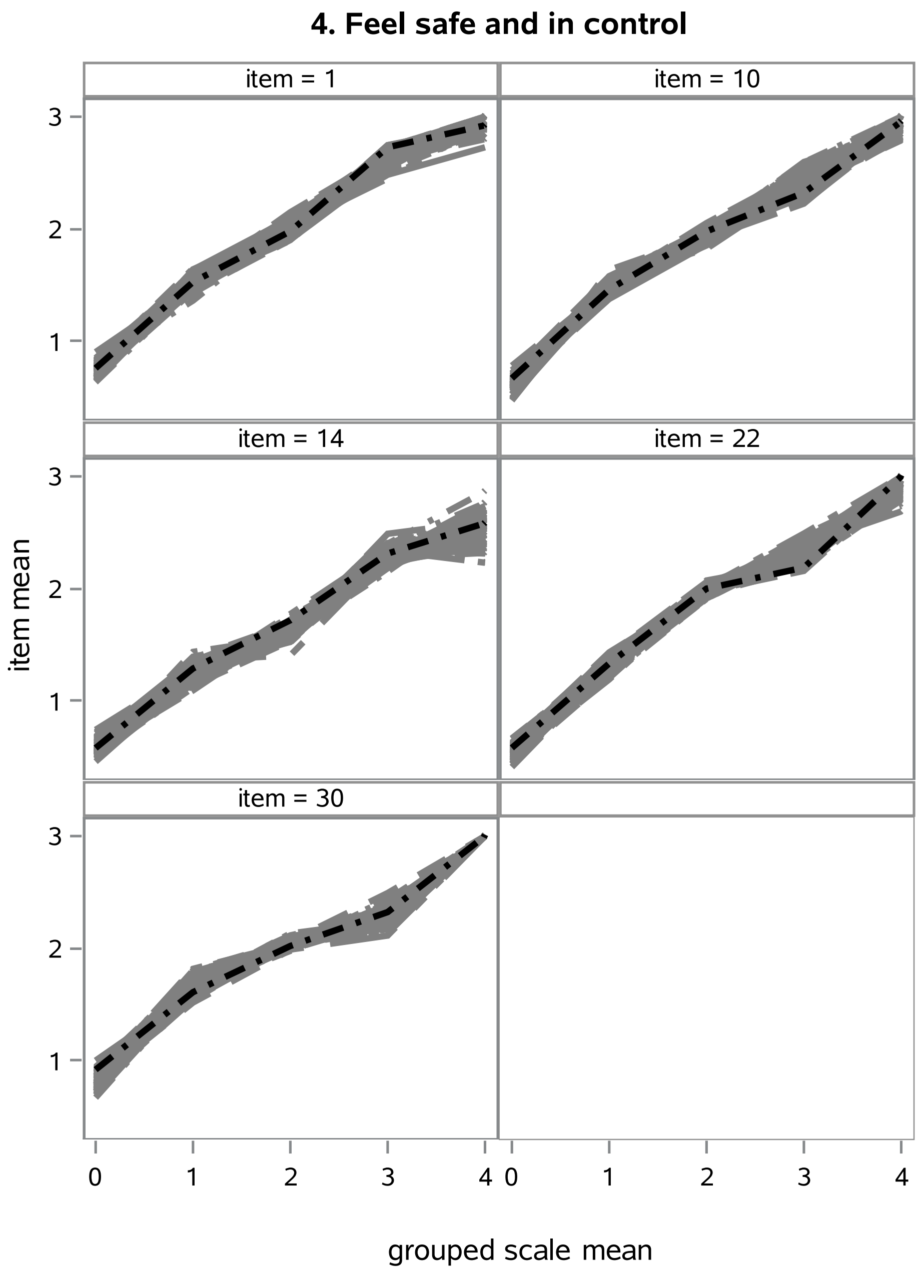
4. Feel safe and in control
item = 1
item = 10
item = 14
item = 22
item = 30
item mean
grouped scale mean
0
1
2
3
4

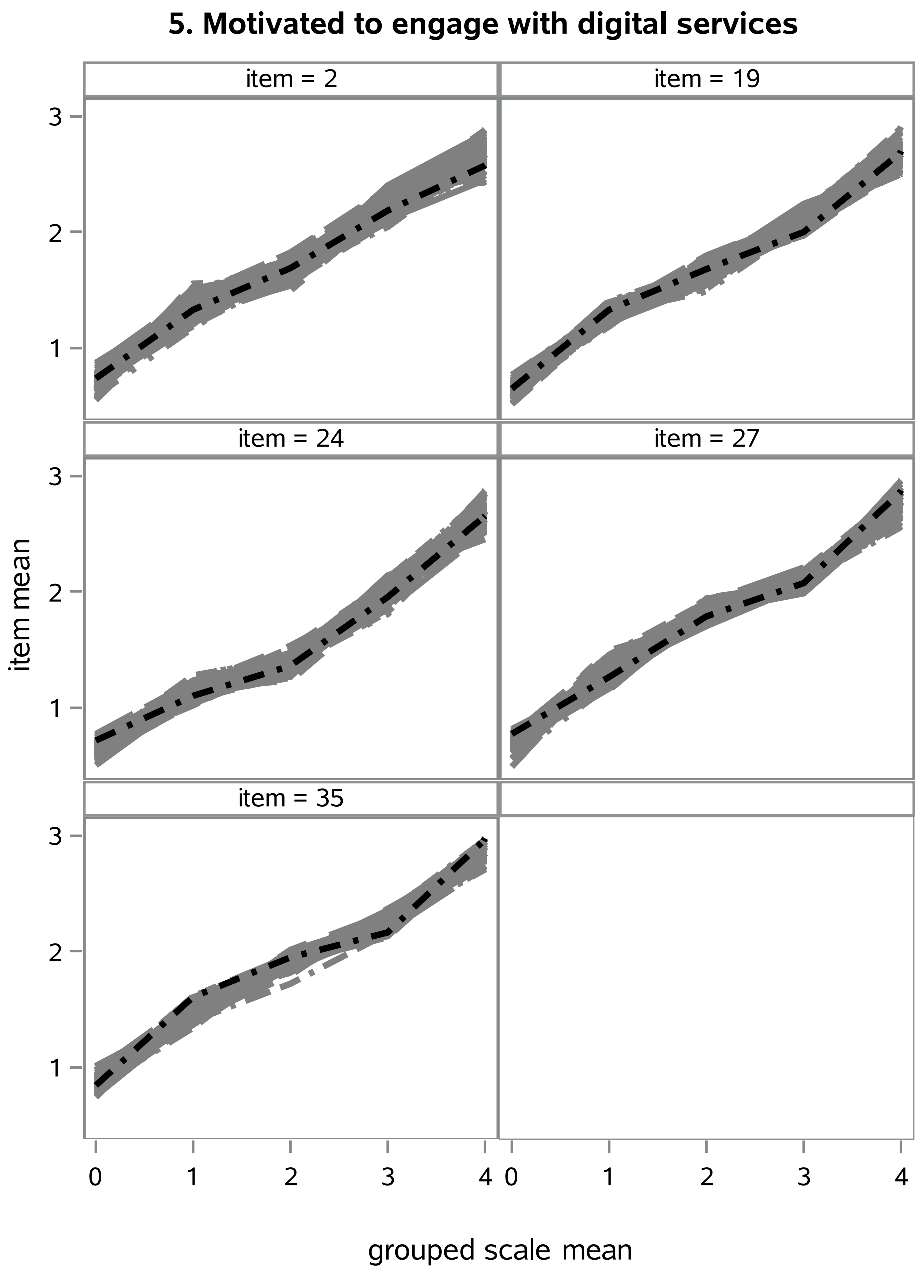
5. Motivated to engage with digital services
item = 2
item = 19
item = 24
item = 27
item = 35
item mean
grouped scale mean
0
1
2
3
4

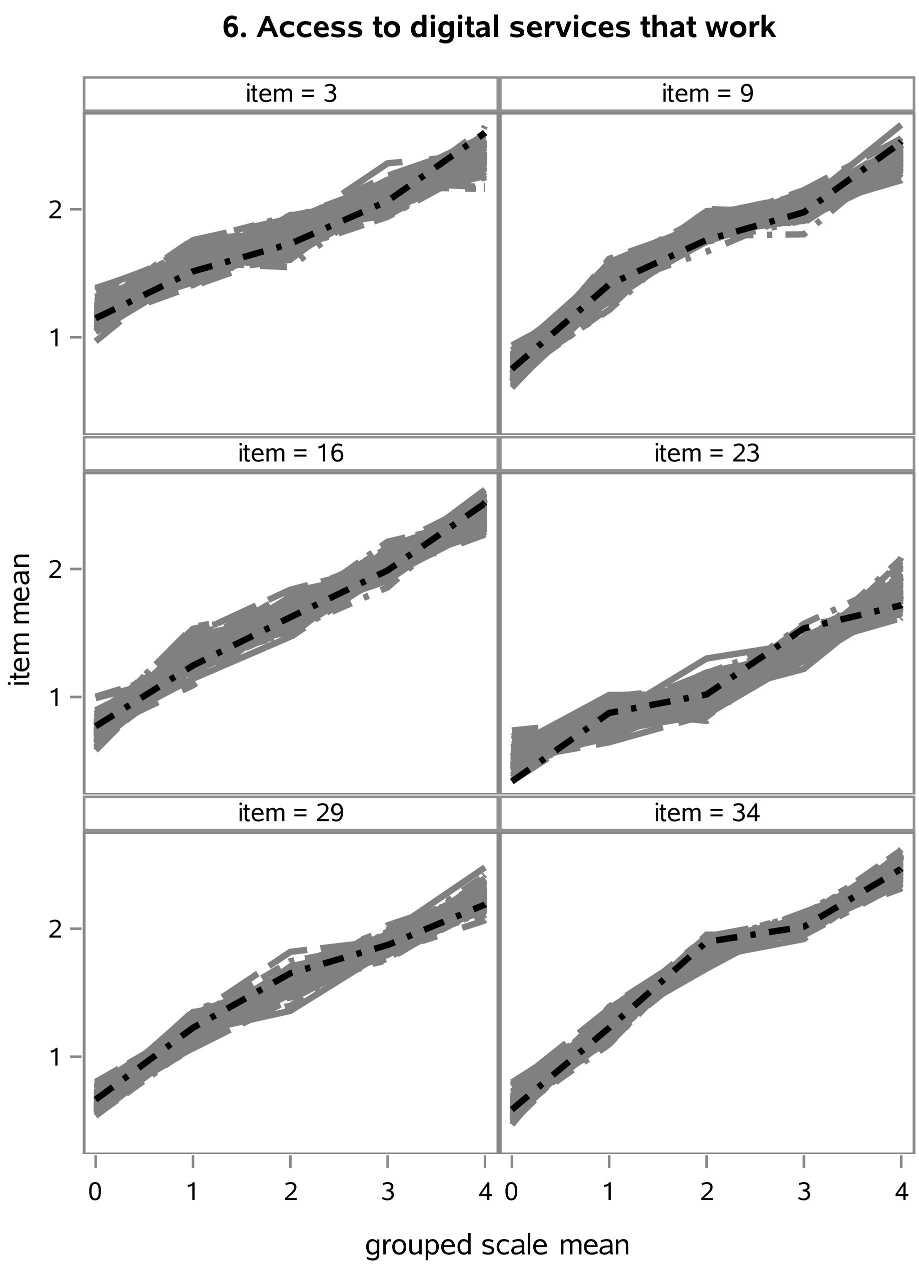
6. Access to digital services that work
item = 3
item = 9
item = 16
item = 23
item = 29
item = 34
item mean
1
2
0
1
2
3
4
grouped scale mean

7. Digital services that suit individual needs

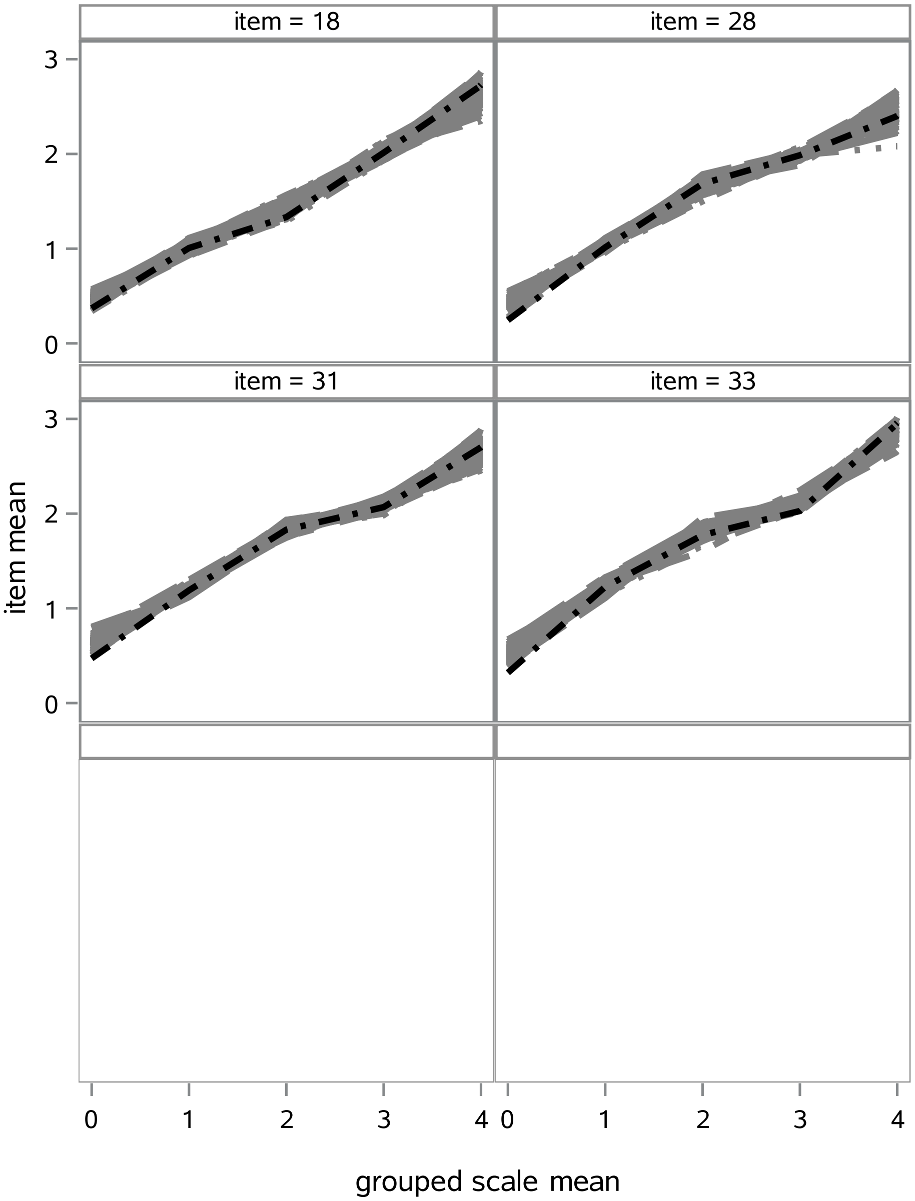

Supplement: Multimedia Appendix 4 [file jmir_v20i2e36_app4.pdf]
