# Supplementary material for: A Multidimensional Tool Based on the eHealth Literacy Framework: Development and Initial Validity Testing of the eHealth Literacy Questionnaire (eHLQ)
Source: J Med Internet Res. 2018 Feb 12;20(2):e36. doi: 10.2196/jmir.8371 (PMC5826975; doi:10.2196/jmir.8371)
Supplement: Multimedia Appendix 5 [file jmir_v20i2e36_app5.pdf]

eHLQ Licence Agreement

Parties

Either of Deakin University and the University of Copenhagen,
as specified in item 1 of the Schedule
(**Licensor)**

The party identified in item 2 of the Schedule
(**Licensee**)

Background

A. Deakin University (**Deakin**) and the University of Copenhagen (**Copenhagen**) jointly own a questionnaire designed to identify and assess levels of e-health literacy (the **eHLQ**).

B. Deakin and Copenhagen have agreed that the eHLQ should be made available for non-commercial use, including non-commercial use by staff and students of academic institutions.

C. The Licensee seeks a licence to eHLQ to enable its use for the Approved Purposes and the Licensor has agreed to provide a licence in respect of eHLQ (**Licence**) to the Licensee on the terms and conditions of this Agreement.

Agreement

1. Definitions and Interpretation

Definitions

In this Agreement:

Approved Purposes means the purposes described in **Item 3** of the **Schedule**.

Commencement Date means the date on which the last of the parties signs this Agreement.

Commercial Use means with respect to the Intellectual Property subsisting in the eHLQ

(a) make, sell, copy, adapt, apply, publish, develop, use, assign, license, sub-license, franchise or otherwise utilise or exploit; or

(b) permit, license, or sublicense a third party to do any of the acts set out in paragraph (a)

for the purpose of generating financial or other commercial gains or benefits (**Commercial Benefit**) (whether or not such Commercial Benefit is ultimately obtained) including:

(c) to use the Intellectual Property to provide a service for which a Commercial Benefit is received;

(d) to use the Intellectual Property to create, or as part of, a product which is, or is to be, sold, hired, leased, distributed or made available to others for Commercial Benefit;

(e) to grant others rights to use the Intellectual Property for their Commercial Benefit .

Data means any information, data, datasets or databases created by or on behalf of a party arising from the administration of the eHLQ.

eHLQ means the e-health literacy questionnaire developed by Lars Kayser, Ole Nørgaard, Dorthe Furstrand Lauritzen, Astrid Karnøe Knudsen and Karl Bang Christensen from the University of Copenhagen and Richard Osborne and Roy Batterham from Deakin University, and includes any materials provided by the Licensor to support the administration of the eHLQ.

Intellectual Property in respect of eHLQ means any and all statutory and other proprietary rights in respect of eHLQ recognised at applicable law including laws relating to trademarks, patents, circuit layout, copyrights, designs, confidential information, know-how and all other rights with respect to Intellectual Property as defined in article 2 of the July 1967 Convention establishing the World Intellectual Property Organisation.

Protocol means a statement prepared with respect to a research project identifying at a minimum, Project title, funding body, names and contact details of investigators, language, rationale, objectives, methods, populations, time frame, expected outcomes, and information about student/ PhD projects.

Term means the period identified in **Item 4** of the **Schedule**, which commences on the Commencement Date and terminates on or before 31 July 2019.

Territory means the territory in which the Licensee is licensed to administer the eHLQ as identified in **Item 5** of the **Schedule**.

Interpretation

1.2 In this Agreement, unless the context requires otherwise, a reference to:

(a) a clause or schedule, is a reference to a clause of or schedule to this Agreement;

(b) ‘this Agreement’ includes any schedules and attachments to this Agreement;

(c) a document or agreement, including this Agreement, includes a reference to that document or agreement as novated, altered or replaced from time to time;

(d) a person, includes a partnership, joint venture, unincorporated association, corporation and a government or statutory body or authority;

(e) ‘dollars’ or ‘$’ is a reference to Australian dollars;

(f) a business day means a day other than a Saturday or Sunday on which banks are open for business in the jurisdiction in which the recipient of a Notice or the party required to perform an obligation or exercise a right is located and if the day on which a thing is to be done under this Agreement is not a business day, it must be done on the next business day;

(g) any law or legislation includes any statutory modification or amendment of that law or legislation and any subordinate legislation or regulations made under that law or legislation;

(h) writing includes typewriting, printing, photocopying and any other method of representing words, figures or symbols in a permanent visible form;

(i) the word ‘include’ or ‘including’ is to be interpreted without limitation;

(j) the singular includes the plural and the plural includes the singular; and

(k) a gender includes all genders.

1.3 If a word or phrase is given a defined meaning, other grammatical forms of that word or phrase have a corresponding meaning.

1.4 Headings are for reference only and do not affect the meaning of this Agreement.

1.5 This Agreement may not be interpreted adversely to a party only because that party was responsible for preparing it.

2. Protocol

2.1 The Licensee acknowledges and agrees that it is a condition of this grant of Licence that the Licensee has provided a Protocol to the Licensor which accurately reflects the use of eHLQ by the Licensee. If any item in the Protocol is changed in a significant or material way at any time during the Term of this Licence, the Licensee will promptly notify the Licensor in writing, including details of such change.

3. Licence

3.1 The Licensor grants to the Licensee a non-exclusive, non-transferable, revocable licence (excluding any right of sub-licence) to use, reproduce and communicate eHLQ for the Approved Purposes in the Territory for the Term, subject to the terms and conditions of this Agreement.

3.2 Under no circumstances may the Licensee make Commercial Use of the eHLQ without the prior written consent of both Deakin and Copenhagen, which may be withheld in the absolute discretion of one or both of them.

3.3 If provided in **Item 3** of the **Schedule**, the Licensor grants to the Licensee the right to use the eHLQ to prepare and produce a cultural adaptation and/or translation of the eHLQ into the language identified in **Item 3** of the **Schedule** (**Translation**) subject to the following conditions:

(a) Licensee must undertake the cultural adaptation and/or translation of the eHLQ only in accordance with the Translation and Cultural Adaptation Procedure attached as **Annexure A**;

(b) Licensee must provide a copy of the forward and backward translations to Deakin and Copenhagen for approval at least 60 days before Licensee proposes to administer the eHLQ (**Administration Date**) to allow sufficient time for review of documents by Deakin and Copenhagen, preparation of the provisional final translation of the eHLQ, local validation, and finalisation as described in **Annexure A.**

(c) Deakin and Copenhagen will jointly own all Intellectual Property rights in the Translation and the Licensee assigns such rights to Deakin and Copenhagen in equal shares upon their creation.

(d) If, with the prior consent of the Licensor, the Licensee engages a third party to prepare the Translation, such third party must assign to Deakin and Copenhagen in writing all Intellectual Property rights in the Translation. The Licensor is entitled to approve the contents of the agreement between the Licensee and third party translator as a condition of providing its consent pursuant to this **clause 3.3(d).**

3.4 The Licensee acknowledges that it may not disclose, use, reproduce, communicate or exploit or permit such disclosure, use, reproduction or communication of eHLQ or any part of eHLQ for any purpose other than the Approved Purposes, or in any jurisdiction other than the Territory, unless otherwise agreed in writing with the Licensor.

Consideration

3.5 The eHLQ is provided to the Licensee on a fee-free basis, however in consideration of the grant of licence to the eHLQ, the Licensee will, at the request of the Licensor, provide to the Licensor de-identified Data arising from the administration of the eHLQ to enable Deakin and Copenhagen to use that Data in further research and in the development, refinement and validation of the eHLQ. Such Data must be provided in English.

4. Obligations of Licensee

4.1 The Licensee must:

(a) obtain all required human research ethics approval prior to first use of the eHLQ for the Approved Purposes

(b) ensure that eHLQ is used only for the Approved Purposes;

(c) reproduce and communicate eHLQ only for the Approved Purposes:

(d) unless provided otherwise as an Approved Purpose in **Item 3** of the **Schedule**, not modify or translate eHLQ, without the express written approval of the Licensor which shall be subject to the conditions set out in **clause 3.3.**

4.2 The Licensee will itself administer eHLQ.

4.3 The Licensee must

(a) treat the eHLQ as confidential and use it only for the purposes of its administration for the Approved Purpose and in compliance with ethical approvals;

(b) protect the eHLQ from unauthorised access, use, modification or disclosure and ensure that it is securely destroyed or deleted at the conclusion of the Term; provided that the Licensee may retain copies of the eHLQ to the extent required to comply with ethics approvals, in which case all copies of the eHLQ shall at all times remain subject to the obligations of confidentiality set out in this Licence Agreement;

(c) ensure that eHLQ is not reprinted or published in its entirety in any publication or presentation; and

(d) ensure that no part of the eHLQ is used in any other questionnaire or similar tool.

4.4 Unless authorised in writing by the Licensor, the Licensee must not, and must not allow or cause any other person to:

(a) print, copy, reproduce or communicate eHLQ by any means or in any form other than for the Approved Purposes during the Term;

(b) give, lease, assign, license, sub-license, transfer, distribute, disclose, disseminate or publish eHLQ in any form to any other person or attempt to do any of these acts; or

(c) alter, change, remove or obscure any notices or other indications (including copyright notices) as to ownership of eHLQ.

5. Intellectual Property

5.1 The Licensee agrees that all Intellectual Property in eHLQ, and any content and/or documentation that accompany and/or are made available through eHLQ, and in any modifications, new versions or enhancements to eHLQ (whether authorised or unauthorised) belongs to Deakin and Copenhagen, and that the only rights the Licensee has in eHLQ are those granted to it under this Agreement.

5.2 The Licensee agrees that if any modifications or adaptations (which, for clarity, include translations) are made to eHLQ by or on behalf of the Licensee or as a consequence of the Licensee's use of eHLQ, all Intellectual Property in such modifications and adaptations must be assigned to Deakin and Copenhagen, and the Licensee will do all things reasonably necessary (including the execution of documentation) to effect such assignment upon request by Deakin and/or Copenhagen.

5.3 The Licensor warrants to the Licensee that it has the right to grant the Licence set out in this Agreement.

6. Warranties and Limitation of Liability

6.1 The Licensee agrees that, to the extent permitted by law, all warranties (including implied warranties), other than the express warranty given in **clause 5.3** of this Agreement, in respect of the subject matter of this Agreement are excluded and of no effect. Where the exclusion of a given implied warranty would be void or unenforceable, the Licensee agrees that Licensor's liability for a breach of such warranty will be limited, at Licensor's discretion to the re-supply of eHLQ or the payment of the cost of the re-supply of eHLQ.

6.2 For the avoidance of doubt, the Licensee agrees that it uses eHLQ entirely at its own risk, and neither Deakin nor Copenhagen warrant that eHLQ is suitable for any particular purpose, that eHLQ will function or perform in a particular manner, or that the Licensee will derive any particular result or outcome from its use of eHLQ.

6.3 The Licensee agrees that Deakin's and Copenhagen's aggregate liability for all causes of action against Deakin and Copenhagen, or either of them whether contractual, tortious or otherwise, will not exceed the aggregate of Licence Fees paid by the Licensee as at the date on which the first such cause of action arose. Neither Deakin nor Copenhagen will be liable to the Licensee for any indirect or consequential losses, damages, costs and/or expenses incurred or sustained by the Licensee under, or as a result of exercising rights in, this Agreement (including as a result of any negligence by Deakin and Copenhagen or either of them), and in particular will not be liable for any loss of revenue or profits, loss of data, loss of goodwill or failure to realise an anticipated saving or benefit.

6.4 The Licensee agrees to indemnify Deakin and Copenhagen from and against liability and all loss and damage of any kind whatsoever caused directly or indirectly by any claim or action against Deakin and Copenhagen, or either of them, arising directly or indirectly out of the Licensee's use of eHLQ or any breach by the Licensee of the terms and conditions of this Agreement.

7. Termination

7.1 The Licensor may terminate this Agreement immediately by giving written notice of termination to the Licensee if the Licensee breaches its obligations in respect of this Agreement and fails to remedy such breach within 14 days of receiving a notice from the Licensor specifying the breach and requesting its rectification.

7.2 Upon termination of this Agreement, all licences granted under this Agreement terminate, and the Licensee must immediately cease all use of eHLQ.

7.3 Termination, completion or expiry of this Agreement for any reason shall not extinguish or otherwise affect:

(a) any rights of either party against the other party which:

(i) accrued prior to the time of the termination, completion or expiry; or

(ii) otherwise relate to or may arise at any future time from any breach or non- observance of obligations under this Agreement which arose prior to the time of the termination, completion or expiry; or

(iii) provisions, which by their nature survive termination, completion or expiry (including **clauses 4, 5, 6, 7.2** and **8**).

8. Confidentiality

8.1 The Licensee undertakes to keep secret and protect the confidential nature of all information and documentation provided to it, learned by it or to which it has or has had access, arising out of or in connection with any aspect of the negotiation or performance of this Agreement including, without limitation, the terms of this Licence Agreement. To this end the Licensee must not use, disclose or in any way communicate to any other person the details of any Confidential Information without the prior written consent of the Licensor.

9. Notices

9.1 A notice, demand, consent or other communication (**Notice**) given to a party under this Agreement is only effective if it is in writing delivered or sent by prepaid post to that party at its address set out in this Agreement.

9.2 Subject to **clause 9.3** a Notice given for any purpose under this Agreement is taken to be received:

(a) if hand delivered, on delivery;

(b) if sent by prepaid post, five (or in the case of a Notice sent to another country, nine) business days after the date of posting;

9.3 If any Notice is given on a day that is not a business day or after 5.00pm on a business day in the place of business of the receiving party, it is to be treated as having been given at the beginning of the next business day.

9.4 If a party gives the other party three business days' notice of a change of its address a Notice is only effective if it is given to that party at the latest address.

10. Dispute Resolution

10.1 Where there is a dispute concerning this Agreement, a party alleging the dispute must serve on the other party a notice setting out the details of the dispute.

10.2 On receipt of a notice under **clause 10.1** senior officers representing each of the parties must meet within five business days and, acting reasonably and in good faith, do their best to resolve the dispute through negotiation.

10.3 Neither party may commence any court proceedings (except for applications for urgent injunctive relief) for a period of 20 days after the meeting referred to in **clause 10.2**.

11. Miscellaneous

Entire Agreement

11.1 This Agreement constitutes the entire agreement between the parties as to its subject matter. It supersedes all prior understandings or agreements between the parties and any prior condition, warranty, indemnity or representation imposed, given or made by a party in connection with that subject matter.

Variation

11.2 This Agreement may only be altered or varied in writing signed by each of the parties.

Waiver

11.3 A waiver of any right under this Agreement must be in writing signed by the party granting it. A waiver is only effective in relation to the particular obligation or breach for which it is given. It is not to be taken as an implied waiver of any other obligation or breach or an implied waiver of that obligation on any other occasion.

11.4 The fact that a party fails to do, or delays in doing, something the party is entitled to do under this Agreement does not amount to a waiver.

Assignments and Transfers

11.5 A party must not assign or transfer any of its rights or obligations under this Agreement without the prior written consent of the other party.

Severability

11.6 Part or all of any clause of this Agreement that is illegal or unenforceable in any jurisdiction will be severed in the relevant jurisdiction and the remaining provisions of this Agreement will continue in force. The legality or enforceability of the provision in any other jurisdiction will not be affected.

Costs

11.7 Except as otherwise set out in this Agreement, each party must pay its own costs and expenses in relation to preparing, negotiating, executing and completing this Agreement and any document related to this Agreement.

Execution of Separate Documents

11.8 This Agreement is properly executed when;

(a) each party executes this document; or

(b) if the parties execute separate but identical documents, when those separately executed documents are exchanged between the parties, including by mail, facsimile transmission or electronically.

Governing Law

11.9 This Agreement is governed by

(a) If Deakin is the Licensor named in **Item 1** of the **Schedule**, the laws of the State of Victoria, Australia;

(b) If Copenhagen is the Licensor named in **Item 1** of the **Schedule**, the laws of Denmark.
